# Supplementary figures and images for: Small G Protein Regulates Virus Infection via MiRNA and Autophagy in Shrimp
Source: Biomolecules. 2025 Feb 13;15(2):277. doi: 10.3390/biom15020277 (PMC11853464; doi:10.3390/biom15020277)

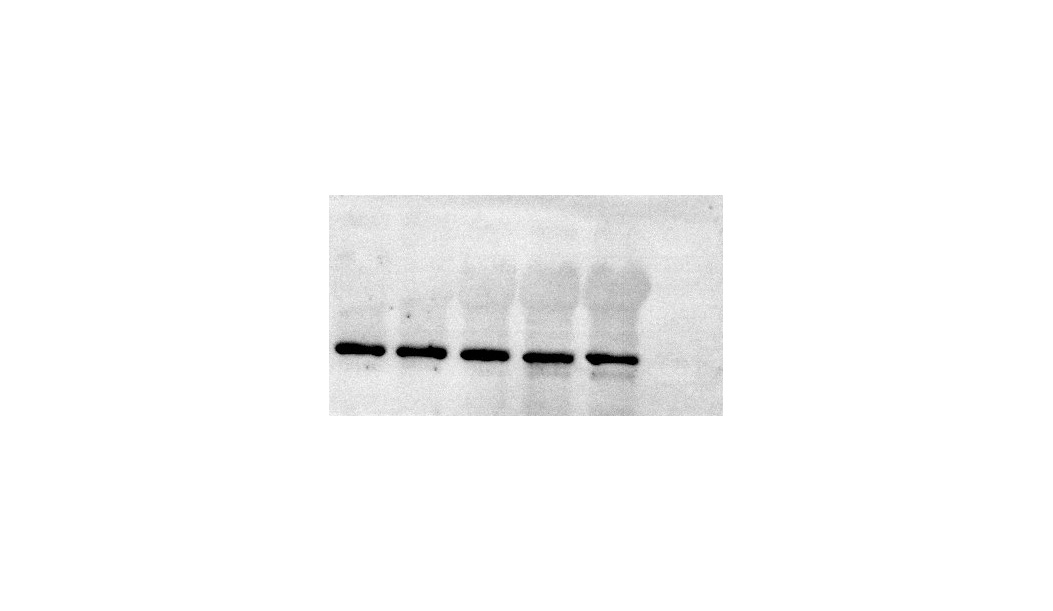

Supplement: Supplementary file 1 [file biomolecules-15-00277-s001.zip › biomolecules-3419574-supplementary/data/figS1/actin-B.jpg]

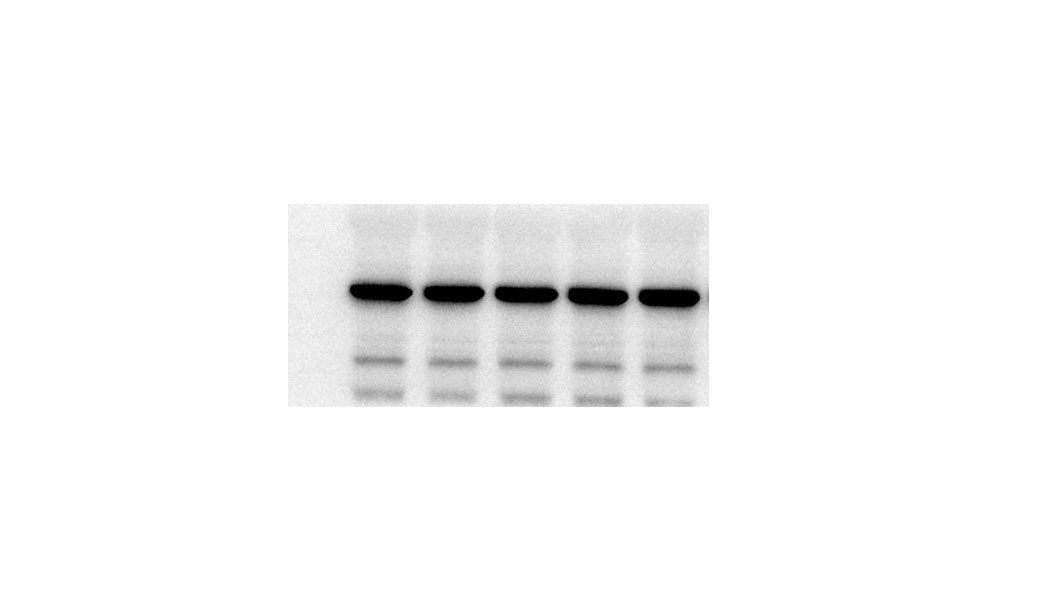

Supplement: Supplementary file 1 [file biomolecules-15-00277-s001.zip › biomolecules-3419574-supplementary/data/figS1/actin-D.jpg]

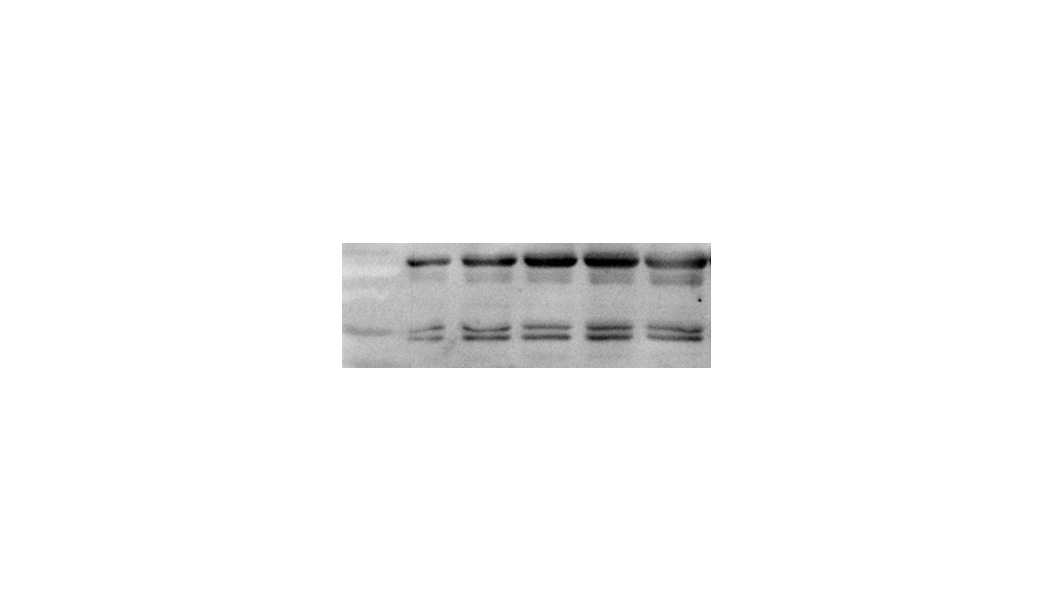

Supplement: Supplementary file 1 [file biomolecules-15-00277-s001.zip › biomolecules-3419574-supplementary/data/figS1/lc3-B.jpg]

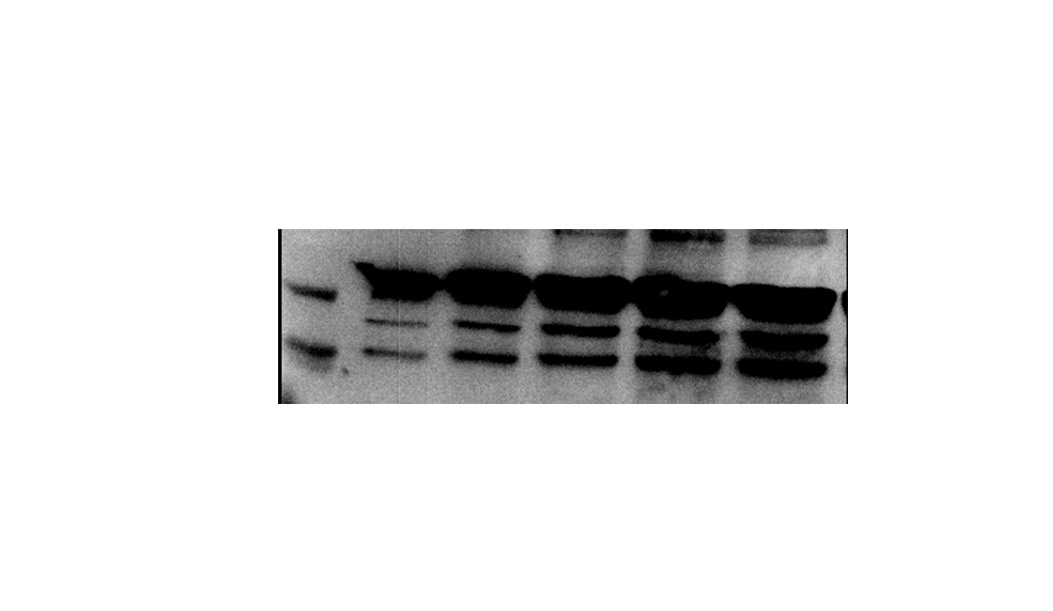

Supplement: Supplementary file 1 [file biomolecules-15-00277-s001.zip › biomolecules-3419574-supplementary/data/figS1/rab10-B.jpg]

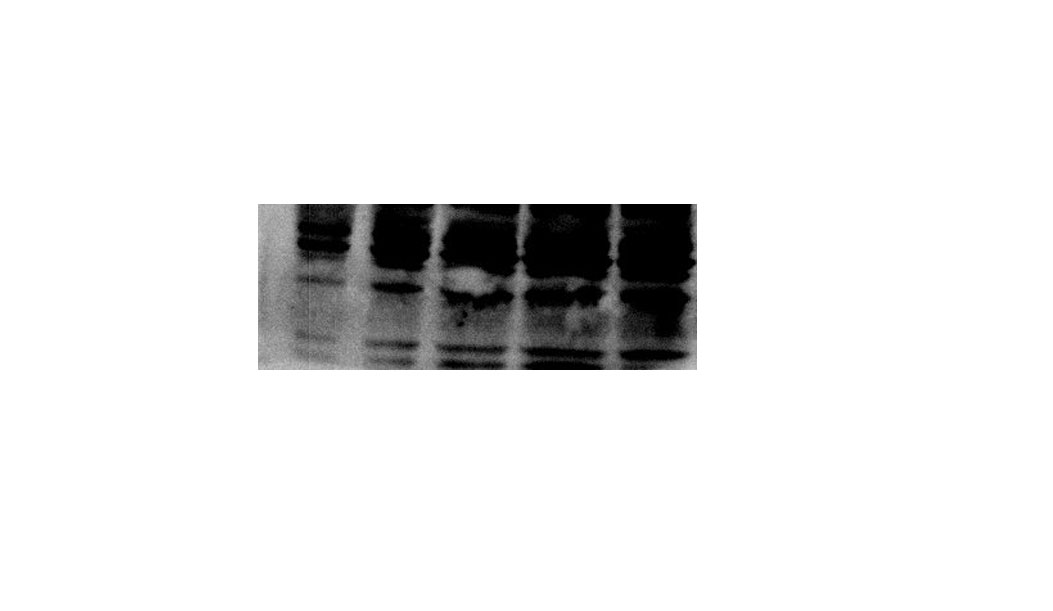

Supplement: Supplementary file 1 [file biomolecules-15-00277-s001.zip › biomolecules-3419574-supplementary/data/figS1/rab10-D.jpg]

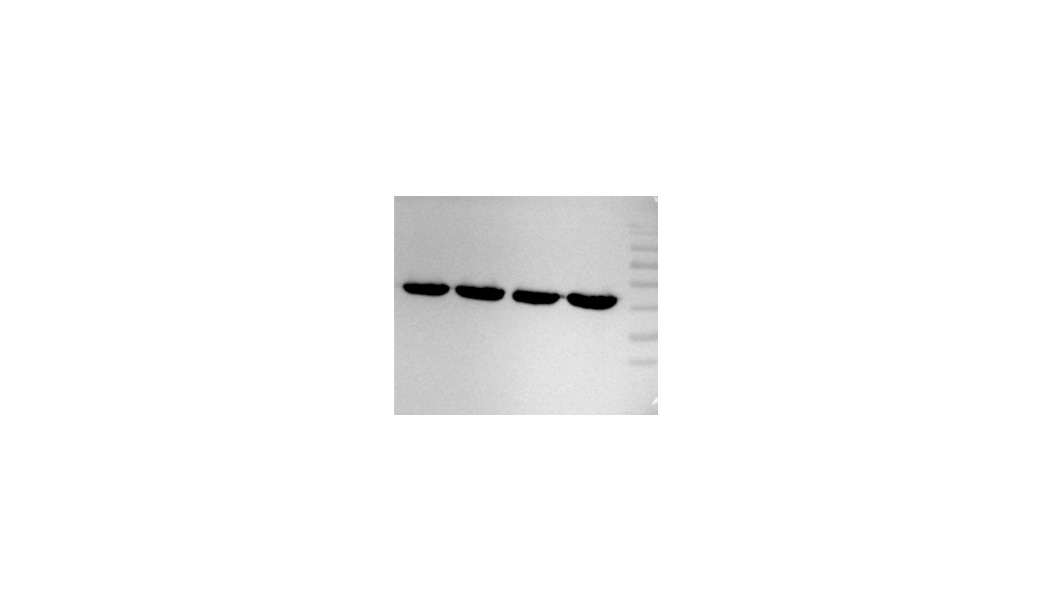

Supplement: Supplementary file 1 [file biomolecules-15-00277-s001.zip › biomolecules-3419574-supplementary/data/FigS2/actin-B.jpg]

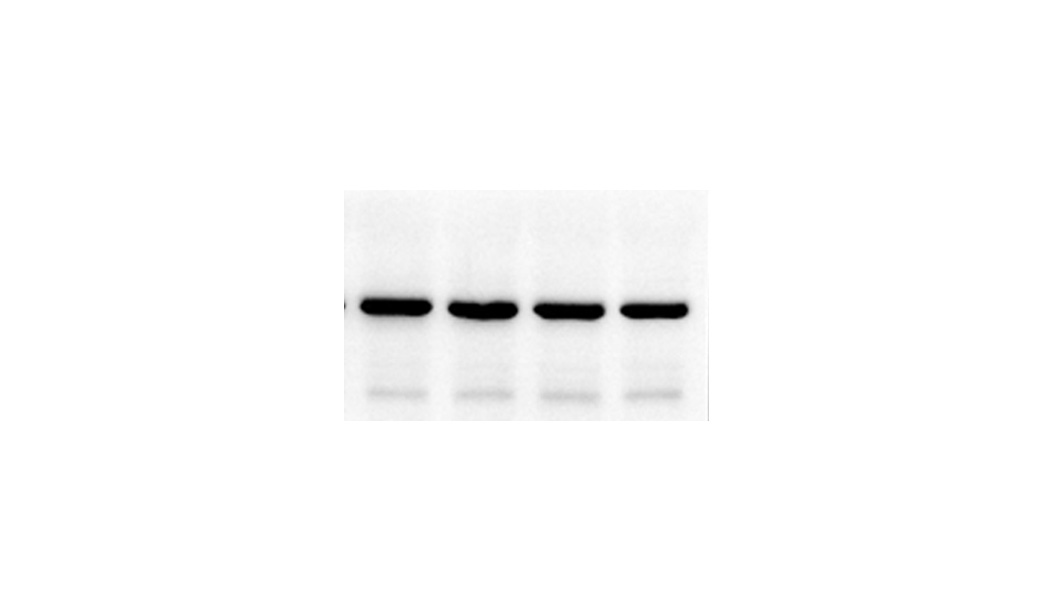

Supplement: Supplementary file 1 [file biomolecules-15-00277-s001.zip › biomolecules-3419574-supplementary/data/FigS2/actin-D.jpg]

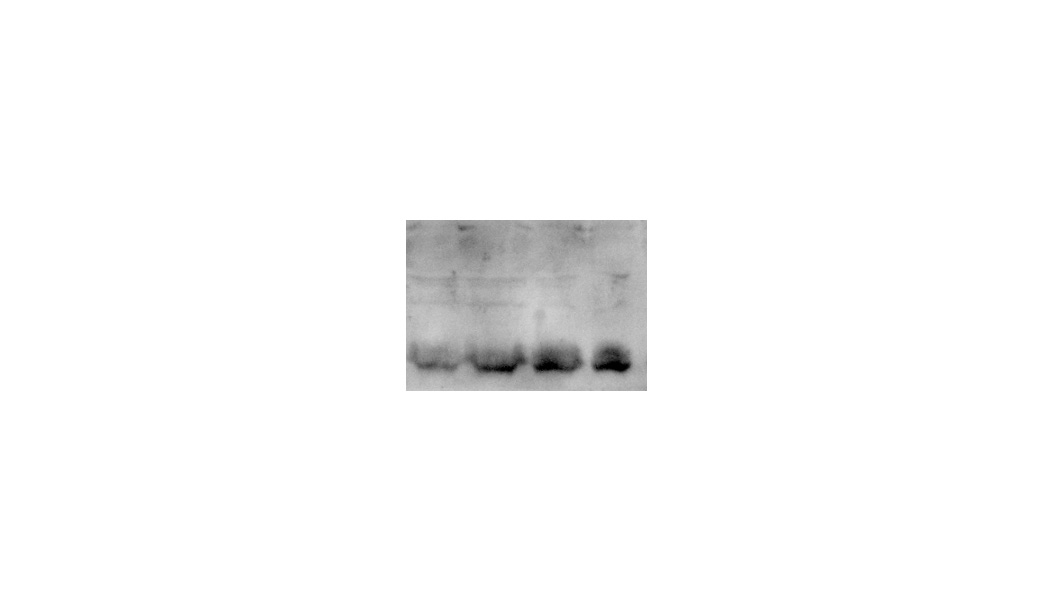

Supplement: Supplementary file 1 [file biomolecules-15-00277-s001.zip › biomolecules-3419574-supplementary/data/FigS2/rab10-B.jpg]

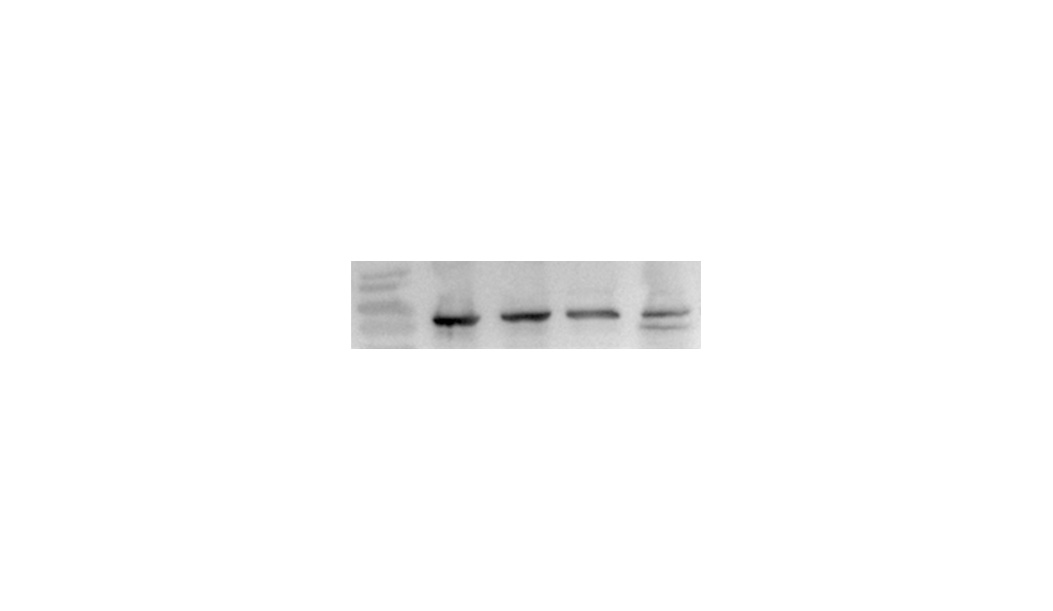

Supplement: Supplementary file 1 [file biomolecules-15-00277-s001.zip › biomolecules-3419574-supplementary/data/FigS2/Rab10-D.jpg]

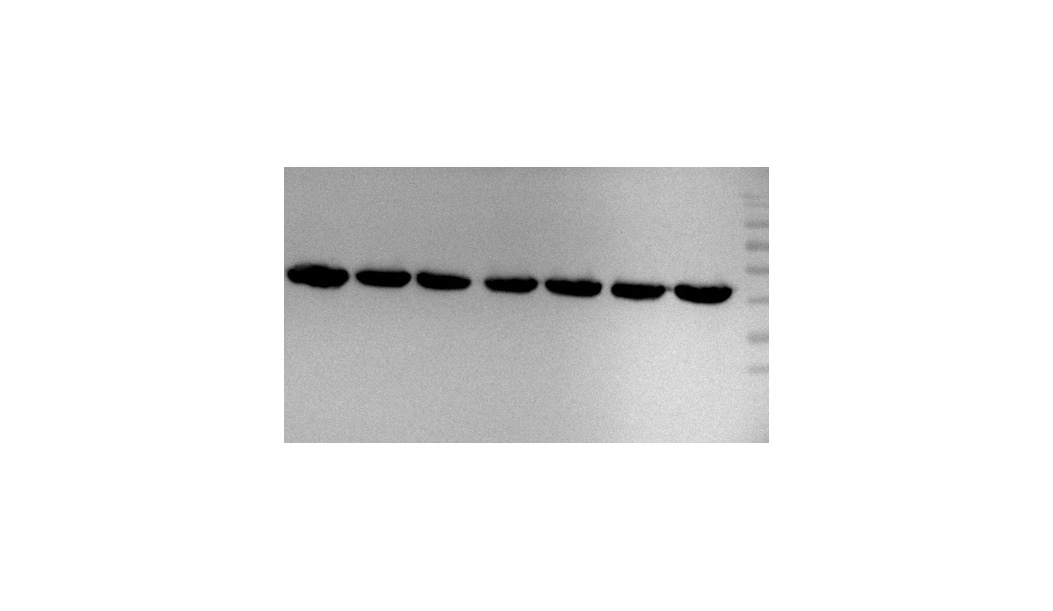

Supplement: Supplementary file 1 [file biomolecules-15-00277-s001.zip › biomolecules-3419574-supplementary/data/figS3/actin-F.jpg]

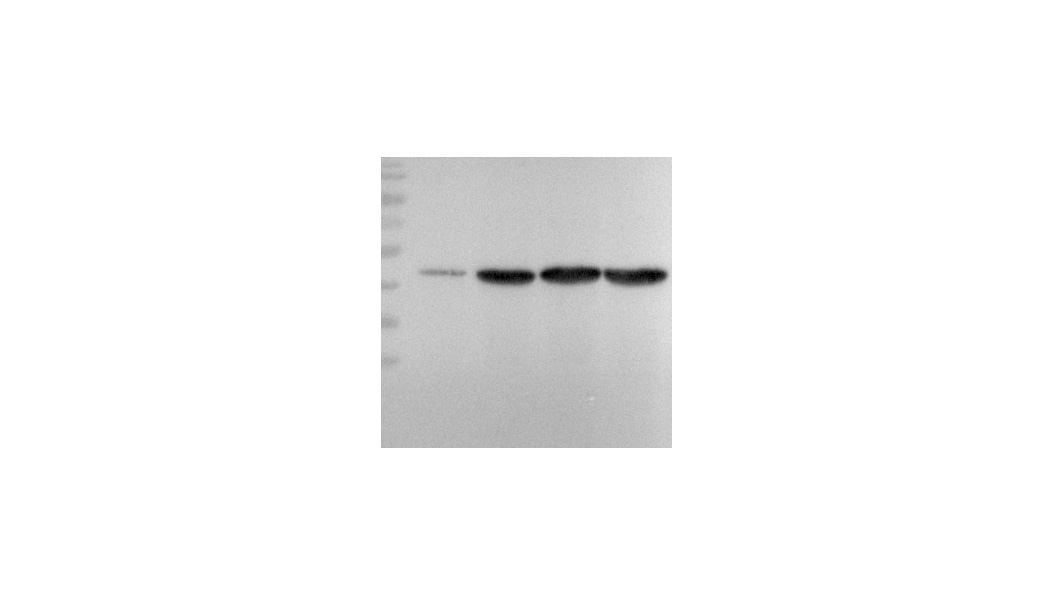

Supplement: Supplementary file 1 [file biomolecules-15-00277-s001.zip › biomolecules-3419574-supplementary/data/figS3/actin-H.jpg]

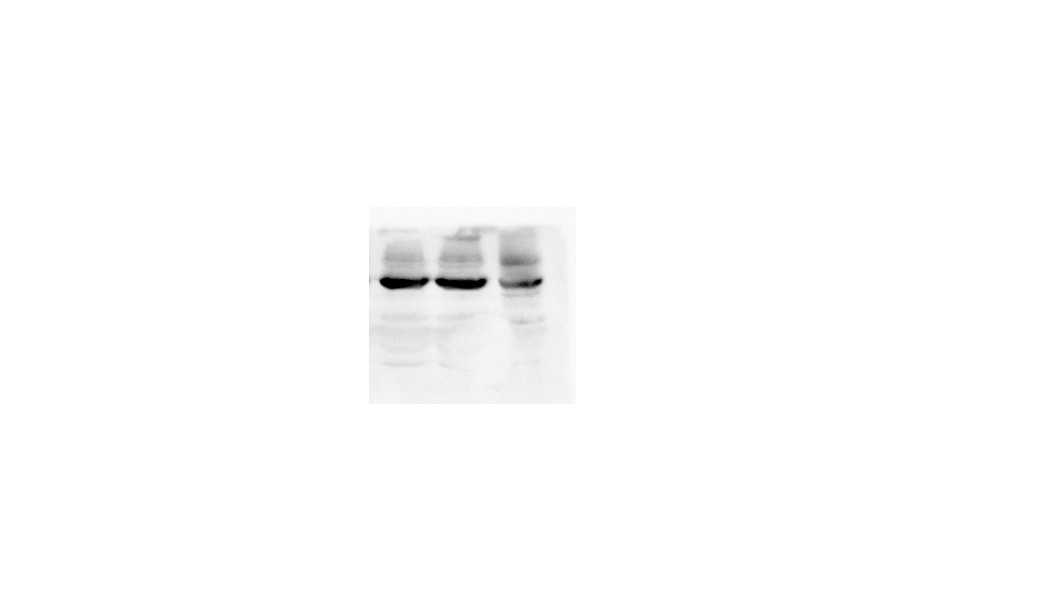

Supplement: Supplementary file 1 [file biomolecules-15-00277-s001.zip › biomolecules-3419574-supplementary/data/figS3/rab10-F.jpg]

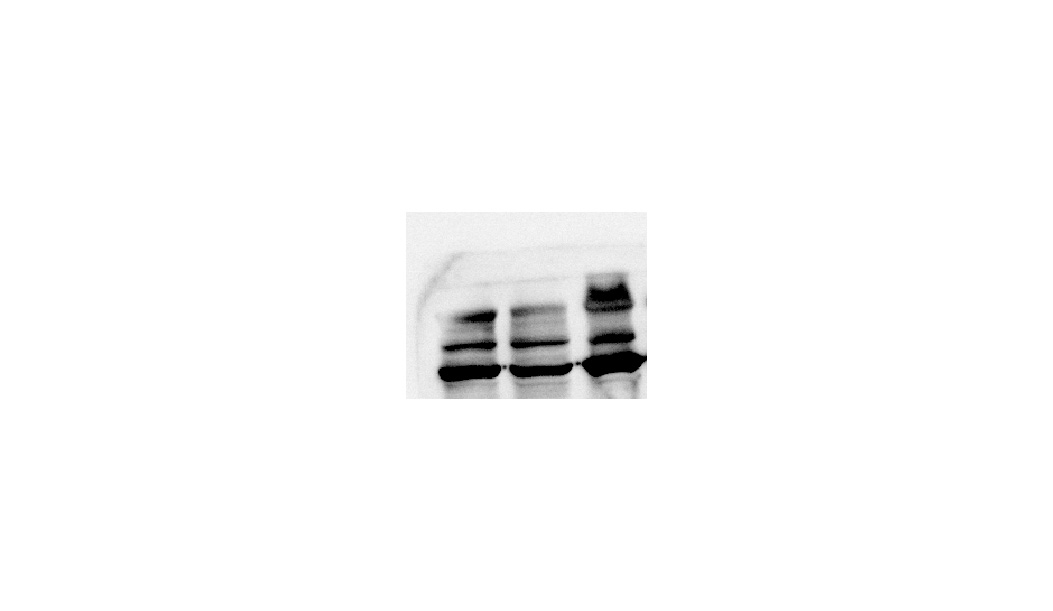

Supplement: Supplementary file 1 [file biomolecules-15-00277-s001.zip › biomolecules-3419574-supplementary/data/figS3/rab10-H.jpg]

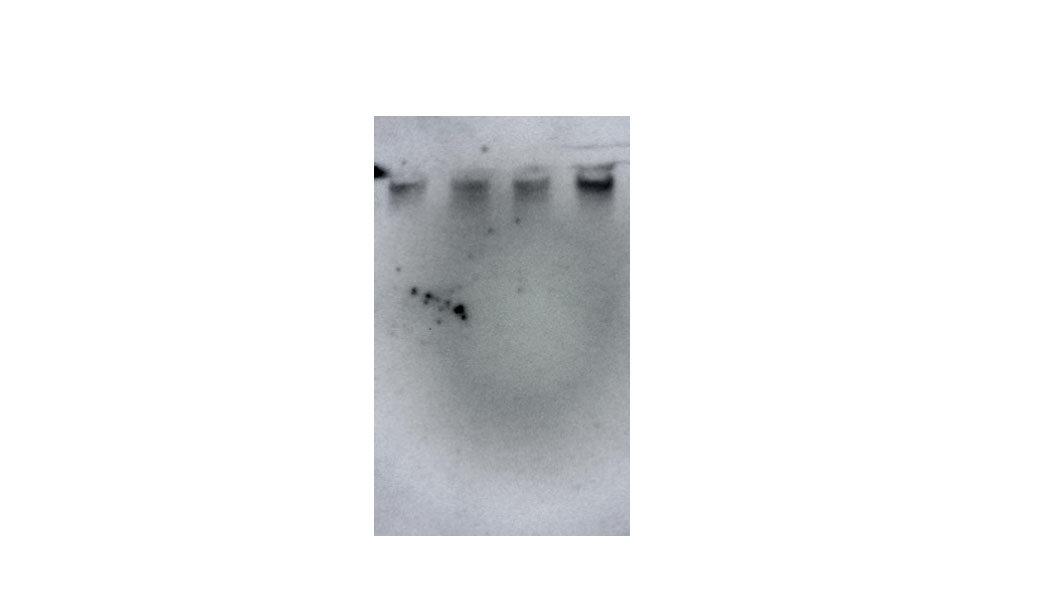

Supplement: Supplementary file 1 [file biomolecules-15-00277-s001.zip › biomolecules-3419574-supplementary/data/fig-S4/miR2c-A.jpg]

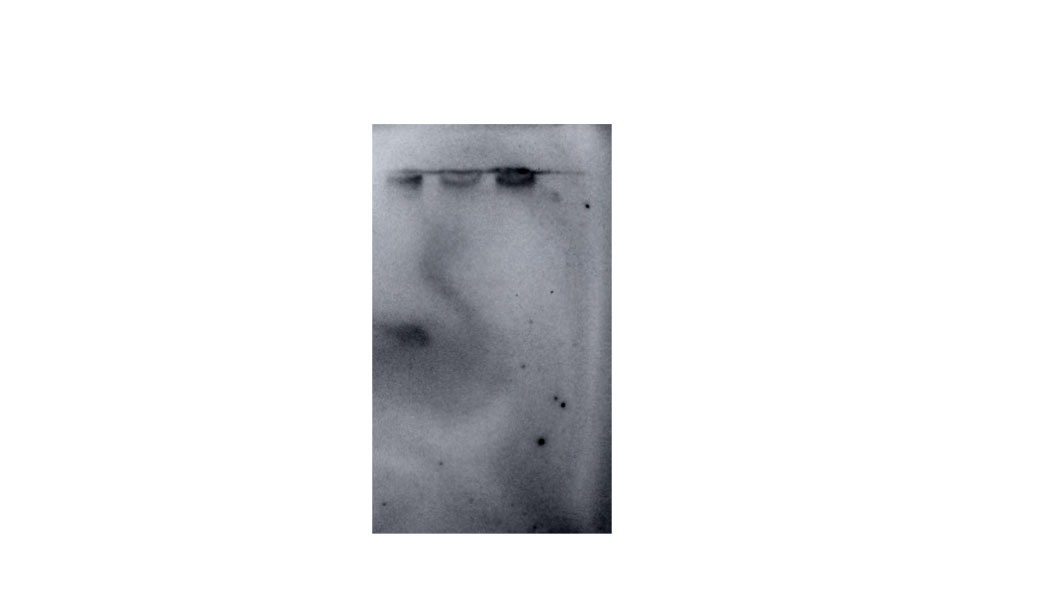

Supplement: Supplementary file 1 [file biomolecules-15-00277-s001.zip › biomolecules-3419574-supplementary/data/fig-S4/miR2c-B.jpg]

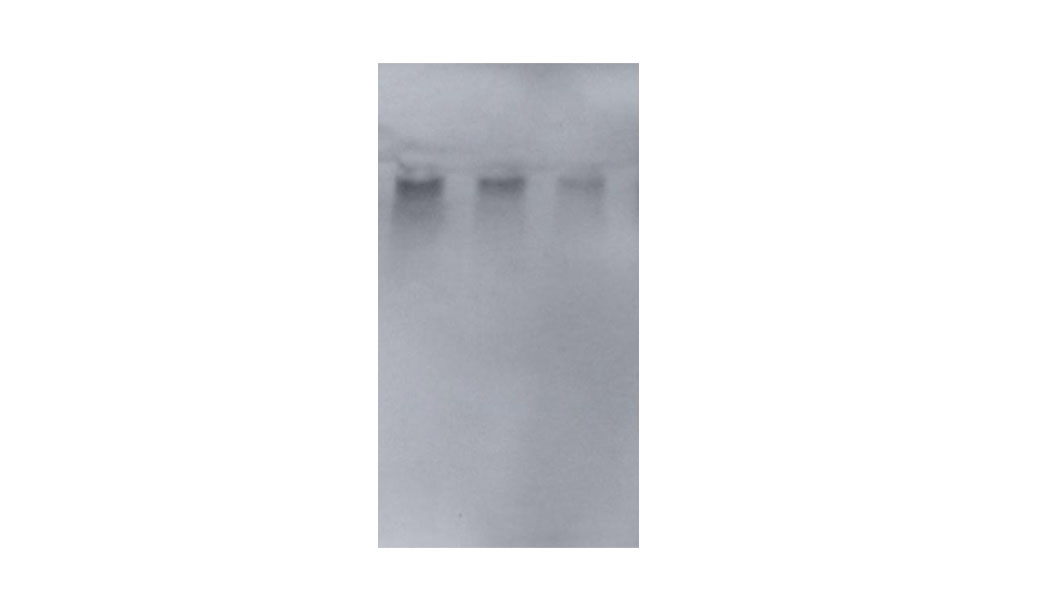

Supplement: Supplementary file 1 [file biomolecules-15-00277-s001.zip › biomolecules-3419574-supplementary/data/fig-S4/miR2c-E.jpg]

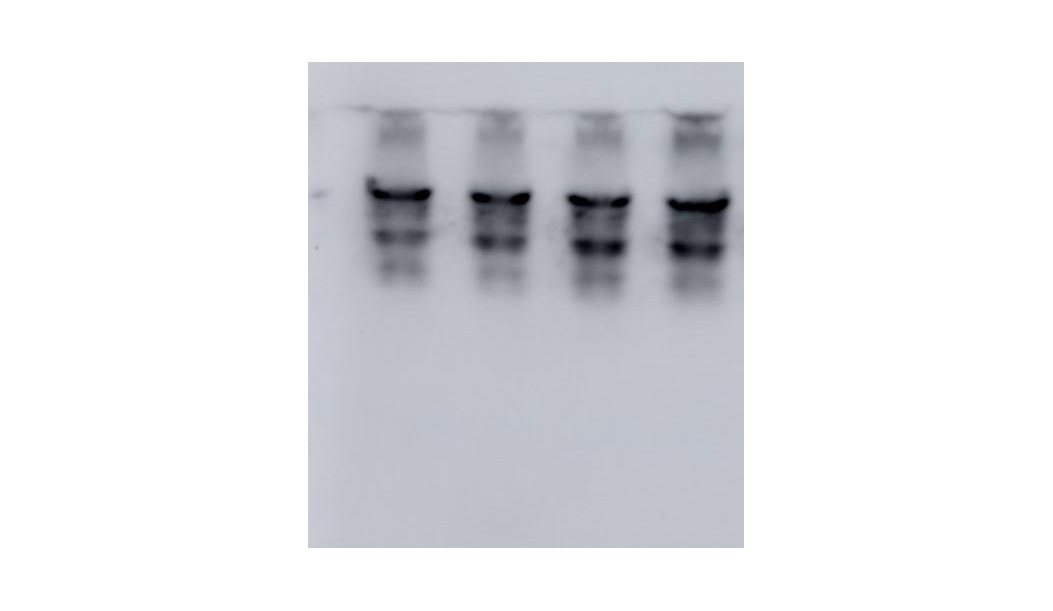

Supplement: Supplementary file 1 [file biomolecules-15-00277-s001.zip › biomolecules-3419574-supplementary/data/fig-S4/u6-A.jpg]

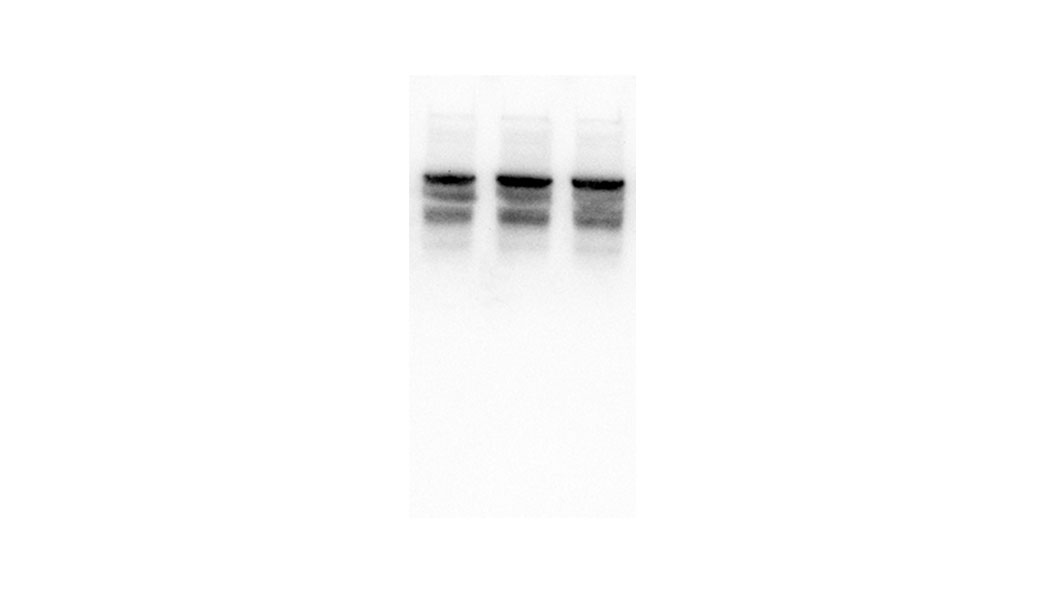

Supplement: Supplementary file 1 [file biomolecules-15-00277-s001.zip › biomolecules-3419574-supplementary/data/fig-S4/U6-B.jpg]

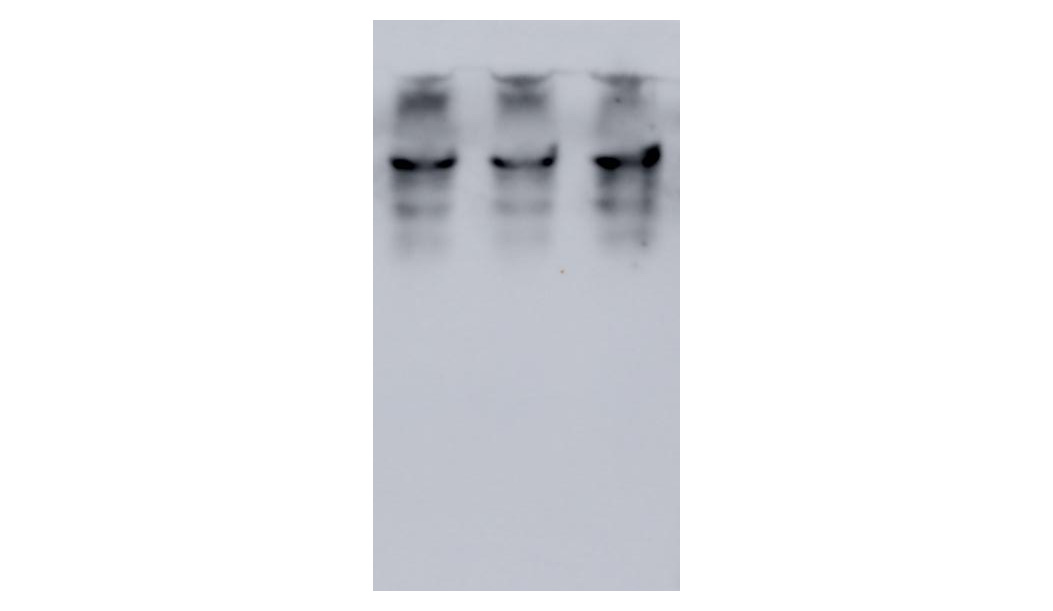

Supplement: Supplementary file 1 [file biomolecules-15-00277-s001.zip › biomolecules-3419574-supplementary/data/fig-S4/U6-E.jpg]
